# The role of oxygen and water on molybdenum nanoclusters for electro catalytic ammonia production

**DOI:** 10.3762/bjnano.5.11

**Published:** 2014-01-31

**Authors:** Jakob G Howalt, Tejs Vegge

**Affiliations:** 1 Department of Energy Conversion and Storage, Technical University of Denmark, DK-4000 Roskilde, Denmark; 2 Center for Atomic-scale Materials Design, Department of Physics, Technical University of Denmark, DK-2800 Kgs. Lyngby, Denmark

**Keywords:** ammonia, density functional theory, electrocatalysis, nanoparticles, oxygen poisoning

## Abstract

The presence of water often gives rise to oxygen adsorption on catalyst surfaces through decomposition of water and the adsorbed oxygen or hydroxide species often occupy important surfaces sites, resulting in a decrease or a total hindrance of other chemical reactions taking place at that site. In this study, we present theoretical investigations of the influence of oxygen adsorption and reduction on pure and nitrogen covered molybdenum nanocluster electro catalysts for electrochemical reduction of N_2_ to NH_3_ with the purpose of understanding oxygen and water poisoning of the catalyst. Density functional theory calculations are used in combination with the computational hydrogen electrode approach to calculate the free energy profile for electrochemical protonation of O and N_2_ species on cuboctahedral Mo_13_ nanoclusters. The calculations show that the molybdenum nanocluster will preferentially bind oxygen over nitrogen and hydrogen at neutral bias, but under electrochemical reaction conditions needed for nitrogen reduction, oxygen adsorption is severely weakened and the adsorption energy is comparable to hydrogen and nitrogen adsorption. The potentials required to reduce oxygen off the surface are −0.72 V or lower for all oxygen coverages studied, and it is thus possible to (re)activate (partially) oxidized nanoclusters for electrochemical ammonia production, e.g., using a dry proton conductor or an aqueous electrolyte. At lower oxygen coverages, nitrogen molecules can adsorb to the surface and electrochemical ammonia production via the associative mechanism is possible at potentials as low as −0.45 V to −0.7 V.

## Introduction

Molybdenum nanoclusters have been identified as a prime candidate for electrochemical ammonia production with seemingly low Faradaic losses to hydrogen evolution [1–2]. To produce ammonia electrochemically, one can either use a liquid or a solid electrolyte, but these effectively require wet conditions to obtain sufficient protonic conduction [3–4]. The presence of water may give rise to oxygen or hydroxide adsorption on the surface, which can occupy or block important surfaces sites. The adsorbed oxygen species can either decrease or totally hinder other chemical reactions taking place at that site. Oxygen poisoning of the surface is indeed a main inhibitor for ammonia production [5–6]. In this paper, the presence of oxygen species, e.g., resulting from a dehydrogenation reaction of residual water from a solid electrolyte or an aqueous electrolyte, will be investigated to understand the implications it has on the catalytic properties for electrochemical ammonia production. In addition, the blocking of active sites by oxygen species has been explored; together with a determination of reduction pathways to electrochemically reduce the blocking oxygen off the surface.

### Computational Method

#### DFT calculations

The calculations were carried out with density functional theory (DFT) calculations [7–8] using the RPBE exchange correlation functional [9] along with the projector augmented wave method [10–11] as implemented in the GPAW code [12–14]. A grid of (3,3) for the finite difference stencils have been used together with a grid spacing of 0.18 Å and a minimum of 20 free bands above the Fermi level. Periodic boundary conditions with a Monkhorst–Pack [15] k-point sampling of 2 × 2 × 2 were used to perform a methodological consistent comparison with previously obtained results on stepped and flat surfaces [1,16]. A 7 Å vacuum layer on all sides of the nanocluster is applied, giving a separation of 14 Å between the clusters. When solving the electronic density self-consistently, the convergence criteria have been chosen such that the changes were ≤10^−5^ eV for the energy and 10^−4^ electrons per valence electron for the density. In all calculations, a Fermi smearing of 10^−4^ eV has been used. The atomic simulation environment ASE [17] was used to set up the atomic structure of these systems. All calculations were performed without spin polarization in order to enable full structural (and atomic) relaxation of the full Mo_13_ nanocluster together with the adsorbates (N, H, O, NH, etc.); these were carried out using the BFGS and FIRE [18] optimizers within ASE.

#### Electrochemical modelling

The procedure for electrochemical reduction of nitrogen molecules through the associative mechanism is outlined in [2,16]. It was shown that the associative mechanism is the preferred route for electrochemical ammonia production, where the protonation of the nitrogen molecules give rise to a weakened N–N bond and subsequent splitting at the third or fourth protonation step. The dissociative mechanism, the other route for electrochemical ammonia production, is due to high dissociation barriers (1.8 eV) of N_2_ not taking place at the surface.

For the purposes of analyzing the reduction of oxygen, a two-step electron-transfer process was assumed and simulated using the Heyrovsky-type [19] reaction. In an acidic environment, the reaction comprises of these elementary reaction steps:


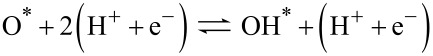



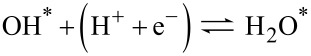



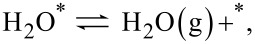


where e^−^ is an electron in the electrode, H^+^ is a proton in the electrolyte and * is a surface site. The reference potential is set to that of the standard hydrogen electrode, and through the computational hydrogen electrode approach the electrons and protons are introduced into the analysis, where 1/2 H_2_ 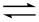 H^+^ + e^−^. Hereby, a description of the effects of an external applied potential *U* on the electrons and the concentration of protons in the electrolyte [20–24] are implemented.

The adsorption energy of O* under electrochemical reaction conditions under an applied potential *U* are calculated as 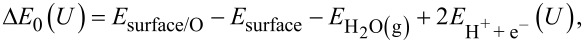 where *E*_surface/O_ and *E*_surface_ are the total energies of the molybdenum nanocluster with and without the specific oxygen atom adsorbed, and 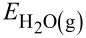 is the gas phase energy of water and 
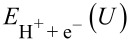
 are the energy of protons and electrons with an applied potential defined by the computational hydrogen electrode approach. Molecular O_2_ is not expected to be present under operating conditions and the sole contributor of O atoms is water. At standard ambient temperature and pressure the adsorption energies are corrected with zero-point energy, *E*_ZPE_, and entropy, TS, contributions at room temperatures.

[1]
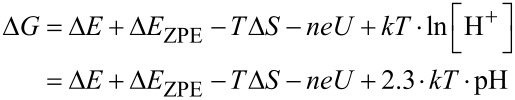


In this paper the pH value is set to 0 and the values of the free energy corrections for all the oxygen containing species were found in literature [25], were the Δ*E*_ZPE_ –*T*Δ*S* corrections for O is 0.05 eV, for OH is 0.35 eV and for H_2_O is 0.67 eV. A pH of, e.g., 7 would change the energy by 0.41 eV for all the reduction mechanisms, leaving the relative activity unchanged.

## Results and Discussion

### Oxygen adsorbed in nitrogen vacancies

**Figure 1:**
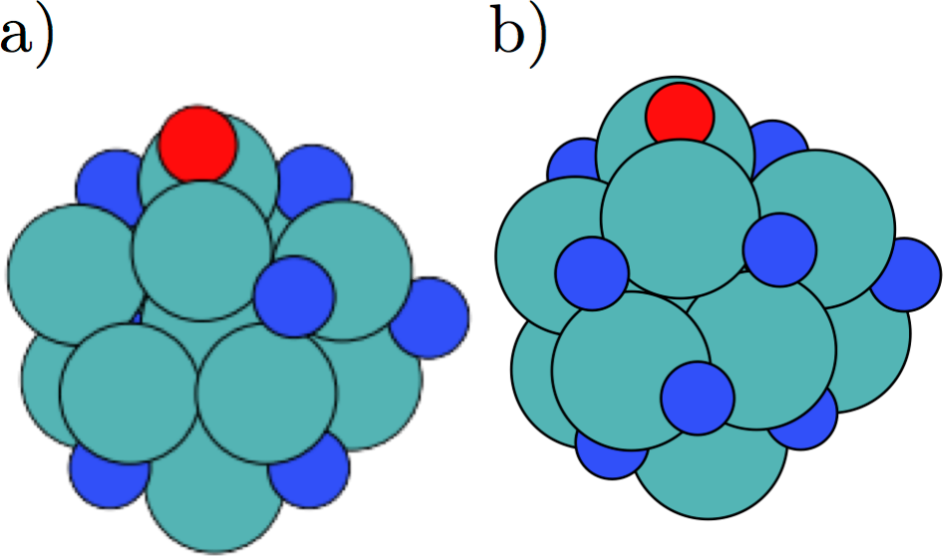
(a) Adsorption of oxygen at a nitrogen vacancy site on Mo_13_N_10_, and (b) adsorption of oxygen at a nitrogen vacancy site on Mo_13_N_12_. Oxygen is adsorbed in the bridge site in both cases.

We have previously shown that partially reduced nitrogen covered molybdenum nanoclusters are promising catalysts for electrochemical production of ammonia [2]. N_2_ adsorption is preferred over H in nitrogen vacancy sites at lower nitrogen coverages at the potential of *U* = −0.6 V needed for electrochemical ammonia production through the associative mechanism [2]. Adsorption of oxygen atoms at nitrogen vacancy sites is presented in Figure 1, where they have been adsorbed at the vacant bridge site.

The free energies for oxygen absorption are shown in Table 1 for neutral bias and the potential needed for ammonia production, and listed together with the energies for nitrogen and hydrogen (from [2]). At neutral bias, oxygen is the preferred adsorbate with adsorption energies of −1.58 eV increasing to −2.15 eV. Under these conditions, the vacancy sites will be filled with oxygen and block nitrogen molecules from adsorbing to the active sites and stop the electrochemical formation of ammonia.

**Table 1:** Stability of nitrogen, hydrogen and oxygen with (*U* = −0.6 V) and without (*U* = 0 V) an applied potential at nitrogen vacancy sites on Mo_13_N_x_ nanoclusters. The potential required to produce ammonia electrochemically at partially nitrogen covered molybdenum nanoclusters was shown to be *U* = −0.6 V [2]. The energies are given with respect to H_2_O (g), H_2_ (g) and N_2_ (g). (The values for Δ*G*_N2_, Δ*G*_H_ (*U* = 0 V) and Δ*G*_H_ (*U* = −0.6 V) are taken from [2]).

	Δ*G*_N2_ [eV]	Δ*G*_H_ [eV] (*U* = 0 V)	Δ*G*_O_ [eV] (*U* = 0 V)	Δ*G*_H_ [eV] (*U* = −0.6 V)	Δ*G*_O_ [eV] (*U* = −0.6 V)

Mo_13_N_12_	−0.06	−0.66	−1.58	−1.26	−0.38
Mo_13_N_11_	−0.43	−0.71	−2.24	−1.31	−1.04
Mo_13_N_10_	−1.2	−0.73	−2.33	−1.33	−1.13
Mo_13_N_9_	−1.13	−0.59	−2.15	−1.19	−0.95

At the potentials needed for ammonia production (−0.6 V), the adsorption of oxygen is weakened by 1.2 eV, due to the change in energy from oxidation of water at the applied potential, i.e., −2*U*. Under these conditions, the adsorption of oxygen has a comparable stability to nitrogen and hydrogen.

The strong preference for oxidation at neutral bias means that the particles will often be (partially) oxidized during synthesis or sample transfer [24]. To free the active sites for N_2_ adsorption and subsequent electrochemical ammonia production, the adsorbed oxygen atoms needs to be removed from the surface sites. One way of re-activating the molybdenum nanocluster for electrochemical ammonia production is therefore to reduce the oxygen electrochemically to water, which will be less strongly bound to the active site and make it possible to bind N_2_ preferentially.

In the case of oxygen adsorption on the more nitrogen rich molybdenum nanoclusters, Table 2 shows the required potentials for the two-proton transfer steps in the reduction of oxygen.

**Table 2:** Reduction of oxygen adsorbed on the Mo_13_N_x_ nanocluster.

	*U*_O→OH_ [V]	*U*_OH→H2O_ [V]

Mo_13_N_12_O	−0.35	−1.62
Mo_13_N_11_O	−0.97	−1.25
Mo_13_N_10_O	−1.18	−1.28
Mo_13_N_9_O	−0.72	−1.41

The potentials range from −1.28 to −1.62 V for the second protonation step, where the formation of H_2_O is hindered by the adsorbed nitrogen atoms in the three fold hollow sites, surrounding the adsorption site of O, OH and H_2_O. The high potential needed for the formation of H_2_O is due to the repositioning of the adsorption site of OH (bridge site) to the adsorption site of H_2_O (ontop site), which is structurally hindered by the nearby adsorbed nitrogen atoms. The removal of oxygen from the partially nitrogen covered molybdenum surface and oxygen will therefore constitute a strong blocking of the active sites and subsequently limit the ammonia production rate through the associative mechanism on partially oxidized nitrogen covered molybdenum nanoclusters.

Direct reduction of the residual nitrogen skin will, however, still be possible and the potential will not be influenced by the presence of oxygen and the nitrogen skin will be reduced electrochemically at −0.6 V as shown in [2].

### An oxygen skin

**Figure 2:**
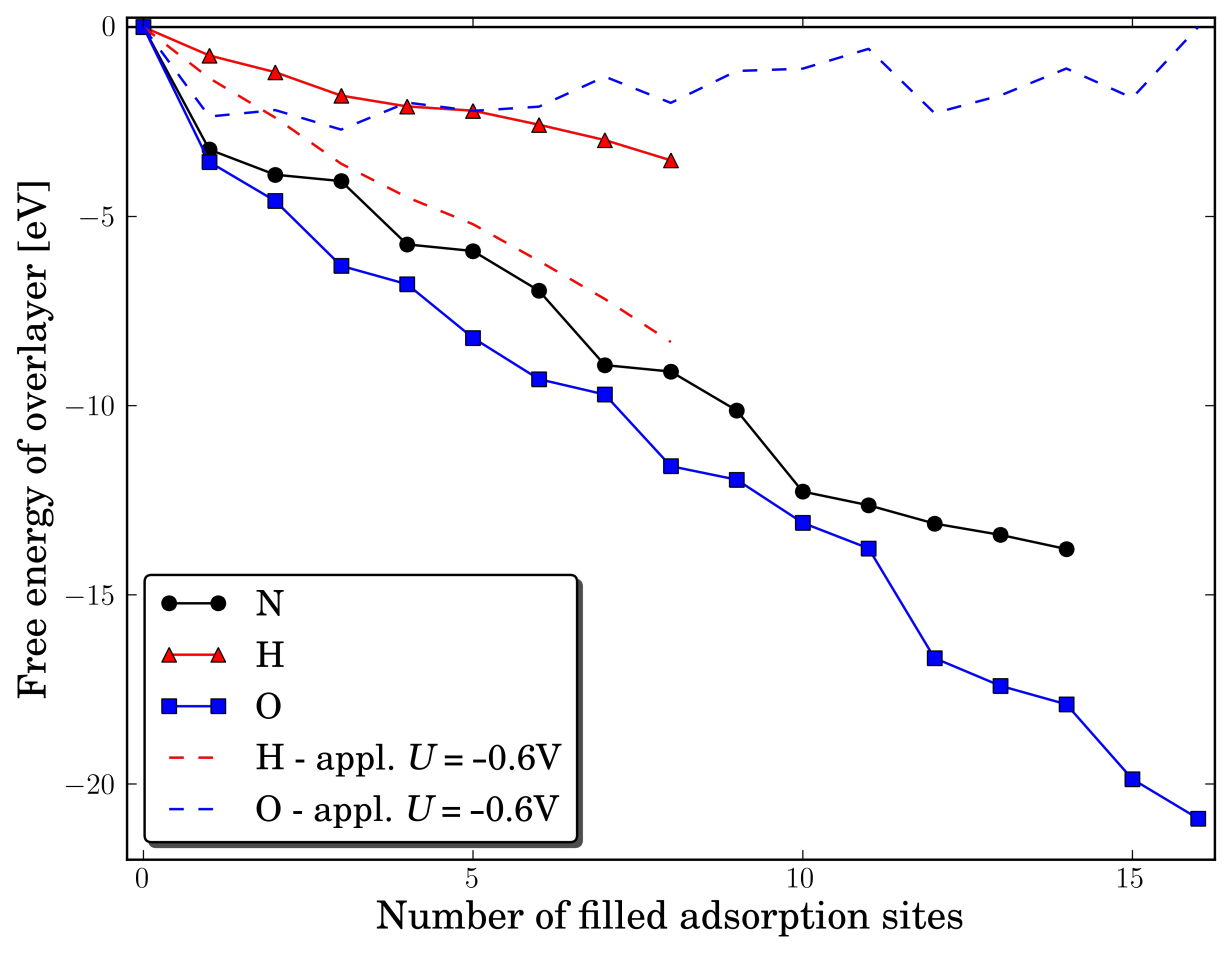
The total free energy for covering the Mo_13_ nanocluster with nitrogen, oxygen or hydrogen. The filled black line shows the filling of the nitrogen skin, while the filled blue line shows the evolution of oxygen coverage at neutral bias. The red coloured line shows the adsorption of hydrogen, while the dashed red coloured line shows adsorption of hydrogen and the dashed blue line shows the adsorption of oxygen at an external applied potential of −0.6 V, which is the potential needed for electrochemically ammonia production previously determined for the clean and nitrogen covered molybdenum nanocluster [2].

The electrochemical production of ammonia will not only occur on nitrogen covered molybdenum clusters, but could also take place at very low or no nitrogen coverage [2]. A clean molybdenum nanocluster in contact with nitrogen, hydrogen and water at neutral bias will also adsorb oxygen from water on the surface, see Figure 2. The figure shows the total adsorption free energies of oxygen, nitrogen and hydrogen as the coverage evolves and until saturation of oxygen and nitrogen is obtained on the surface. This oxygen skin is approximate 1–2 eV more energetically favoured than a nitrogen skin at low coverage. At higher coverage, the oxygen skin becomes even more energetically favoured. However, an applied potential of *U* = −0.6 V, destabilizes oxygen (dashed blue line) significantly with respect to nitrogen and hydrogen (dashed red line), such that an overlayer of oxygen arising from electrochemical splitting of water should not be expected.

A cyclic pattern is seen in the binding energy for both nitrogen and oxygen adatoms, and the distance between the cycles correspond roughly to an addition of 10 electrons, corresponding to the filling of a d-shell. Figure S1 in Supporting Information File 1 shows the d-band of the molybdenum nanocluster as the oxygen coverage increases and it is observed that the d-band broadens and the energetically lower lying d-orbitals are filled additionally as the coverage of oxygen is increased.

**Figure 3:**
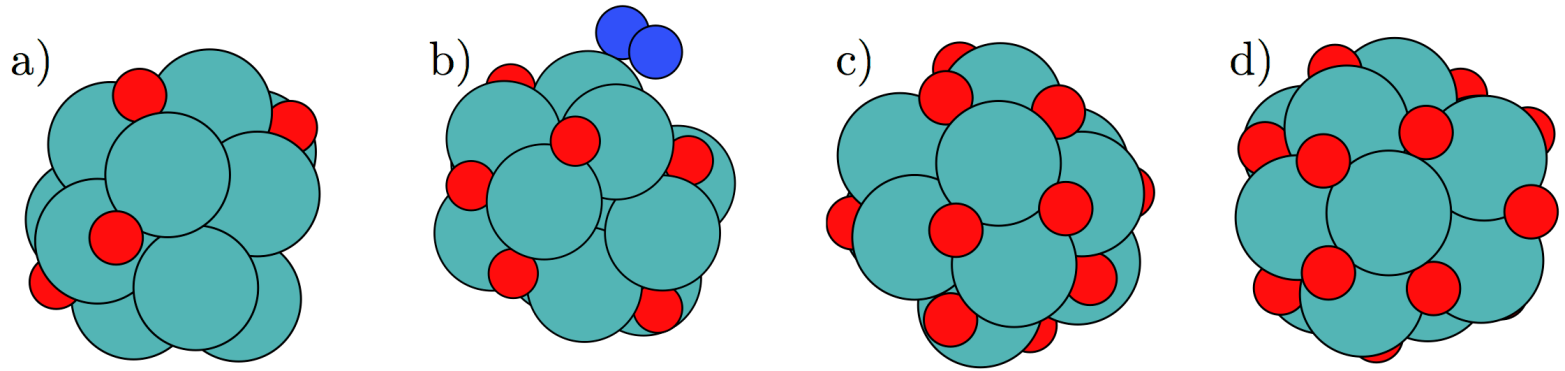
(a) The Mo_13_O_6_ nanocluster, (b) the Mo_13_O_9_ nanocluster with N_2_ adsorbed, (c) the Mo_13_O_12_ with an almost filled oxygen skin, (d) the Mo_13_O_16_ with a filled oxygen skin. At the Mo_13_O_16_, the oxygen adsorption sites are a mix of three fold hollow sites and bridge sites.

The preferred adsorption sites for the oxygen atoms are the three fold hollow sites, see Figure 3a. When all the three fold sites are filled, the oxygen atoms will adsorb in the four fold hollow sites, see Figure 3c, where additional adsorption of a few oxygen atoms greatly distorts the surface; this restructuring allows a higher filling of oxygen on the surface, see Figure 3d. The maximum filling of the surface increases to 16 oxygen atoms and further additions of oxygen atoms are energetically unfavourable. Close to a full overlayer, the binding sites of oxygen becomes asymmetrically and the oxygen atoms now binds in a mix of a three fold hollow site and a bridge site.

### Reduction of the oxygen overlayer

For a fully oxygen covered molybdenum nanocluster, nitrogen molecules cannot adsorb to the surface. Therefore, a full oxygen skin eliminates electrochemical ammonia production. It is therefore necessary to reduce the surface, in order to create active sites for nitrogen adsorption. To investigate the reduction of oxygen on a partially or fully oxygen covered molybdenum nanocluster, the reduction of all adsorbed oxygen atoms should, in principle, be analysed. This is, however, not computationally feasible and instead up to three representative adsorbed oxygen atoms are reduced at each investigated oxygen coverage. Each of the selected oxygen atoms represents a unique oxygen adsorption position and the coverage ranges from 16 oxygen atoms to only one oxygen atom. The potentials needed for the two proton transfer processes are presented in Table 3. For other proton transfer processes (not shown), the local geometries around the adsorption site can hinder both protonation steps, but most often the second protonation is hindered. One such hindrance can be the movements from the adsorption site of the OH species to an on top site, where the H_2_O species is energetically most stable. Such movement requires a restructuring of the local environment, and hence the protonation step can become strongly endothermic. The largest variation is therefore seen for the second protonation step. For the presented reduction steps of OH to H_2_O, the required potentials are in the range of −0.2 to −0.7 V. The formation of OH from O will require a negative potential to stabilize the OH specie compared to the O atom, and in most cases the potential required are in the range of −0.3 V to −0.7 V.

**Table 3:** Reduction of an oxygen overlayer on molybdenum nanoparticles. Multiple adsorbed oxygen atoms have been reduced at the studied oxygen coverage. This is done to probe the reduction of all the unique adsorption sites at the studied oxygen coverage.

	*U*_O→OH_ [V]	*U*_OH→H2O_ [V]

Mo_13_O_16_	−0.49	−0.44
Mo_13_O_14_	0.04	−0.47
Mo_13_O_12_	−0.64	−0.66
Mo_13_O_9_	−0.27	−0.67
Mo_13_O_6_	−0.29	−0.24
Mo_13_O_2_	−0.44	−0.31
Mo_13_O	−0.67	−0.72

For most of the studied coverages, the reaction free energies for desorption of H_2_O off the surface are either low or exergonic. In general, desorption energies are lower than 0.4 eV, but for a single adsorbed H_2_O molecule, it is as high as 1.2 eV. Water should therefore desorb thermally from the surface, except at very low oxygen coverages.

The study of oxygen reduction produces adsorption energies for O, OH, H_2_O at different coverages. No apparent correlation is found between the adsorption energies of *E*_O_, *E*_OH_ and *E*_H2O_ on the cluster, see Figure S2 and Figure S3 in Supporting Information File 1, in contrast to what has previously been observed on metal surfaces, where scaling relations are applicable for OH species on close-packed and stepped surfaces for low coverages [26]. The close packed and stepped surfaces typically have less restructuring during the adsorption of O, OH and H_2_O.

On the molybdenum nanocluster surface, larger local restructuring takes place when either O is added or removed and when either O or OH is protonated. The restructuring of the molybdenum cluster involves all atoms in the nanocluster. This is an effect of the small size of the nanocluster, where the local impact from an adsorption or a reduction step influences the whole nanocluster.

The lowest required potential for water formation at the different coverages is in the range of −0.3 V to −0.7 V. These values are lower or comparable to the required potential needed to form ammonia on the molybdenum nanocluster [2]. Overall, it seems that it is possible to reduce oxygen of the surface at moderate potential of −0.72 V. The potential required for reduction of a surface oxygen atom indicates that the nanoclusters can be reactivated after exposure to water from the electrolyte or the fabrication process.

### Formation of ammonia at relative high oxygen coverage

Nitrogen molecules are not able to adsorb to neither an oxygen vacancy site nor ontop of an oxygen atom at high oxygen coverages. Once the oxygen skin has been partially reduced, the nitrogen molecules adsorb onto the surface at the oxygen vacancy sites, see Figure 3b. The adsorption of nitrogen become stable at a coverage of less than 10 oxygen atoms, see Table 4. The adsorption energies of N_2_ range from −1.0 eV to −1.8 eV depending on the oxygen coverage. The corresponding hydrogen adsorption energies are lower, ranging from −0.65 eV to −0.86 eV, and nitrogen molecules are therefore preferred over hydrogen on the surface at neutral bias. At an applied potential of −0.6 V, which is the potential shown to electrochemically produce ammonia on the clean molybdenum nanocluster, the reaction free energy of adsorbing either a hydrogen atom (coming from H^+^ and e^−^) or a nitrogen molecule will at certain coverages be in favour of H (Mo_13_O_8_ and Mo_13_O_6_) and others of N_2_ (Mo_13_O_9_ and Mo_13_O_7_).

**Table 4:** The adsorption free energies of nitrogen and hydrogen with and without an applied potential on a partly oxygen covered Mo_13_ nanocluster.

	∆*G*_N2_ [eV]	∆*G*_H_ [eV] (*U* = 0 V)	∆*G*_H_ [eV] (*U* = −0.6 V)

Mo_13_O_12_	No binding	−0.23	−0.83
Mo_13_O_10_	0.33	−0.84	−1.44
Mo_13_O_9_	−1.82	−0.72	−1.32
Mo_13_O_8_	−1.02	−0.75	−1.35
Mo_13_O_7_	−1.77	−0.86	−1.46
Mo_13_O_6_	−0.93	−0.65	−1.25

**Figure 4:**
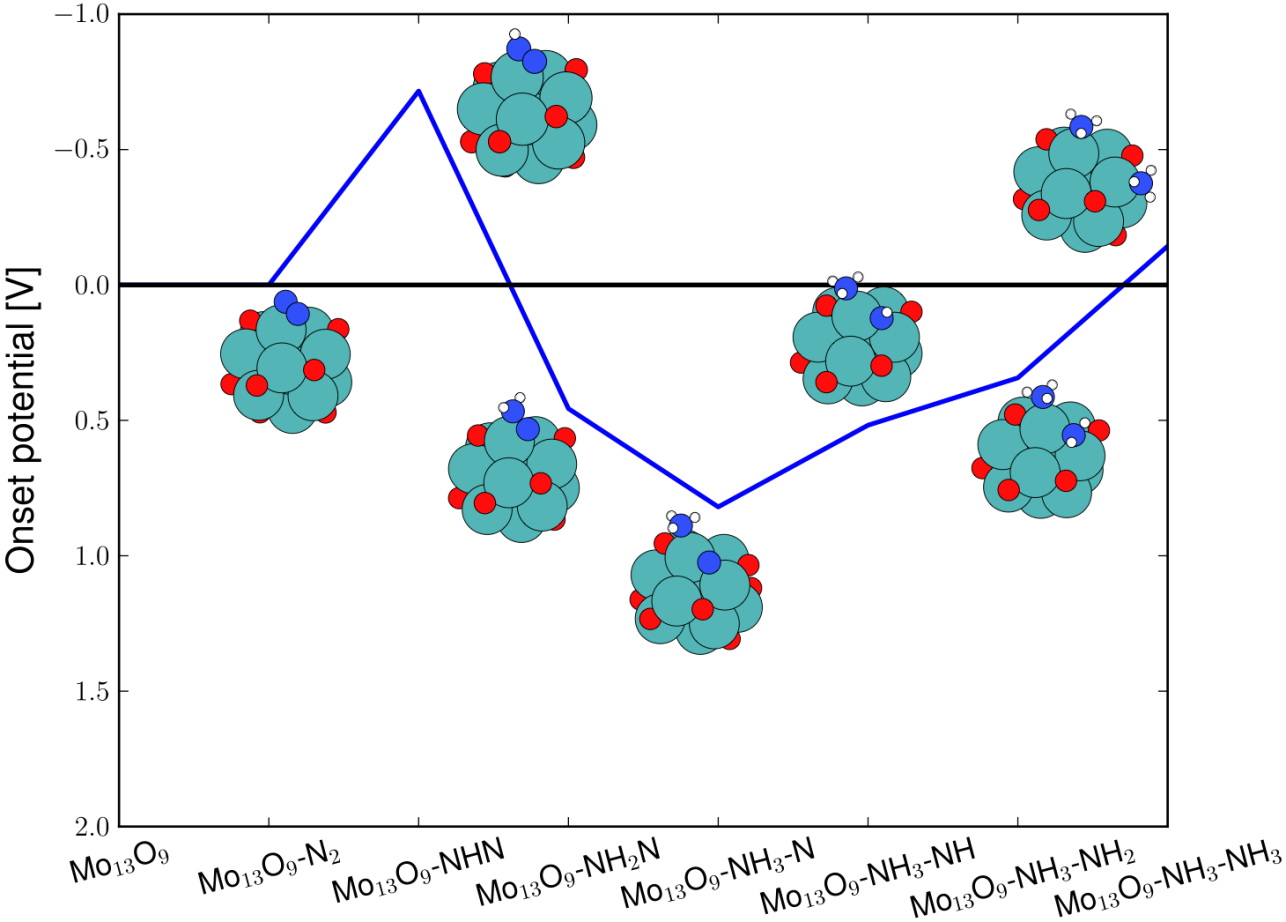
Diagram of the required applied potential to make each reaction step exergonic for electrochemical ammonia production on the Mo_13_O_9_ through the associative mechanism. For all five studied reaction pathways, the highest required potential is −0.7 V for the first protonation step.

For nitrogen molecules adsorbed at a vacancy site on either Mo_13_O_9_ or Mo_13_O_6_, the potential for driving the electrochemical production of ammonia has been determined. These nanoclusters were selected to describe the two regimes with either strong or weak N_2_ adsorption compared to hydrogen adsorption, respectively. On the Mo_13_O_9_ nanocluster, N_2_ is bound most strongly at an applied potential of −0.6 V, while hydrogen is bound stronger on the Mo_13_O_6_ nanocluster. Investigations of the associative pathway on the Mo_13_O_9_ nanocluster, shows the first protonation step to require an onset potential of −0.7 V similar to that needed to reduce oxygen (see Table 3). The onset potentials are shown in Figure 4 for the following electrochemical reaction steps:


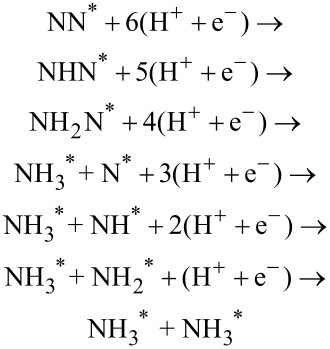


The x-axis show the adsorbed reaction intermediate for each protonation step and NH_2_N denotes a doubly protonated nitrogen molecule with the N–N backbone intact, while NH_3_–NH denotes NH_3_ and NH adsorbed on the surface and the N–N backbone has been dissociated. Five pathways were studied, and all of them had the first initial protonation step to be the potential limiting step with identical required onset potentials.

**Figure 5:**
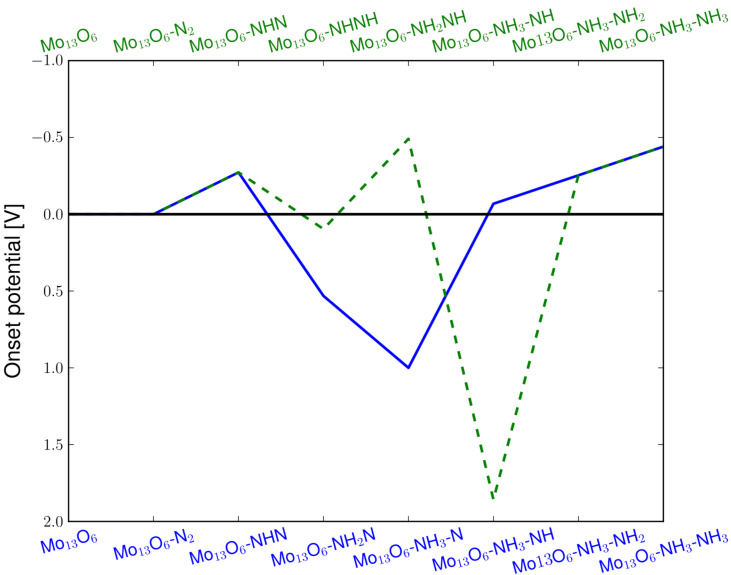
Diagram of the required applied potential to make each reaction step exergonic for electrochemical ammonia production on the Mo_13_O_6_ through the associative mechanism. The limiting reaction is the last protonation to form the second ammonia on the surface requiring a potential of −0.45 V to become exergonic.

For a more reduced oxygen skin, e.g., the Mo_13_O_6_ nanocluster, the energetics of the electrochemical production of ammonia are much more diverse. Here, two routes are very favourably, demanding only potentials of around −0.45 V, to drive the electrochemical production of ammonia, see Figure 5. The routes not shown require potentials of −0.85 V to −1.3 V. The reaction intermediates for the preferred ammonia formation route have been used as the bottom x-axis. The blue filled line on Figure 5 shows that the rate-limiting step is the last protonation; while for the alternative route, marked with the green dashed line and the corresponding reaction intermediates have been used as the top x-axis in Figure 5, the rate-limiting steps are found to be both the third and the last protonation step. The limiting step for this reaction path is the formation of the NH_2_–NH intermediate on the surface. The reaction is then followed by a very exergonic reaction step, where the N–N bond breaks. In both pathways, the N–N bond breaking in the associative process is very exergonic and no apparent activation barrier is observed for the N–N cleavage at either the third or fourth protonation step. The onset potential presented here for electrochemical ammonia production is similar to those obtained in previous studies on both clean and nitrogen covered molybdenum nanoclusters [2].

Instead of protonating the adsorbed nitrogen molecule, the potential could drive the reduction of oxygen. In Table 5, the potentials for driving the oxygen reduction processes are presented. Here, a potential of −0.77 V is required for the high oxygen coverage case, while the last reduction step for the lower oxygen coverage requires significantly larger potentials, i.e., −1.3 V to −1.75 V, to make the reaction exergonic, H_2_O is found to be unstable on Mo_13_O_6_, while Mo_13_O_9_ has a stable adsorption of water.

**Table 5:** The required potentials for reducing oxygen at two oxygen coverages, when nitrogen molecules are present on the partly covered oxygen surfaces.

	*U*_O→OH_ [V]	*U*_OH→H2O_ [V]

Mo_13_O_9_-N_2_	−0.53	−0.77
Mo_13_O_6_-N_2_	−0.26	−1.30

### Competing reaction mechanisms

Here, a possible reaction pathway is presented for electrochemical ammonia production on the Mo_13_O_9_ nanocluster with adsorbed N_2_ at an applied potential *U* = −0.7 V. Three possible mechanisms must be considered: the reduction of N_2_ to ammonia, hydrogen adsorption and evolution, and last the reduction of oxygen and subsequently formation of water. Here, we assume that the adsorption of H with N_2_ present on the surface will be equal to adsorption of H on a surface without N_2_ present.

The reaction free energies of forming H, OH, and N_2_H at *U* = −0.7 V are: ∆*G*[N_2_* + (H^+^ + e^−^) → N_2_H*] = 0 eV, ∆*G*[(H^+^ + e^−^) → H*] = −1.42 eV, ∆*G*[O* + (H^+^ + e^−^) → OH*] = −0.17 eV, respectively. The reaction step with the lowest reaction free energy is hydrogen adsorption on the surface. For adsorbed hydrogen, the next step will be formation of H_2_(g) with a reaction free energies of ∆*G*[H* + (H^+^ + e^−^) → H_2_(g)] = 0.02 eV. The next reaction to occur on the surface will therefore be the reduction of O to OH at −0.17 V. For this reaction intermediate, the next step will produce H_2_O with a reaction free energy of ∆*G*[OH* + (H^+^ + e^-^ ) → H_2_O*] = 0.08 eV. Small energy differences exist for the next protonation step, but N_2_H will be formed on the surface.

The subsequent reaction intermediates for ammonia formation are exergonic at *U* = −0.7 V. The initial production of an overlayer of H is followed by reduction of O into OH, until the electrochemical reduction of N_2_ becomes possible. When the ammonia molecules have desorbed from the surface, adsorption of N_2_ is 0.4 eV more stable than H, which would make the adsorption of nitrogen at a vacancy site energetically favourable. At higher pH-values this difference would increase in favour of N_2_ adsorption because a pH relatively destabilizes the adsorption of H, see Equation 1. Because of the very small difference between the formation of N_2_H, H_2_O and H_2_(g) a Faradaic loss due to hydrogen evolution should be expected and a reduction of the oxygen skin on the surface would also be expected at this oxygen coverage and potential.

For the Mo_13_O_6_ nanocluster with N_2_ adsorbed at an applied potential of *U* = −0.45 V, the preferred mechanism is the following. First hydrogen is bound to the surface, then O is reduced to OH and then finally the reduction of N_2_ can take place. Here, the reaction free energy for production of gas phase hydrogen is ∆*G*[H* + (H^+^ + e^−^) → H_2_(g)] = 0.20 eV and for water formation is ∆*G*[OH + (H^+^ + e^−^) → H_2_O*] = 0.59 eV, while ∆*G* for all electrochemical reaction steps for ammonia production, see Figure 5, are zero or lower as in the previous example on the Mo_13_O_9_ nanocluster.

In the discussion of the preferential reactions on the Mo_13_O_9_ and Mo_13_O_6_ nanoclusters, all possible adsorbate–adsorbate interactions are not included due to the large computational cost associated with a comprehensive investigation. This is a potentially significant assumption, since the adsorption energies may decrease with higher coverage of certain species [27]. For hydrogen evolution on the Mo_13_O_6_ nanocluster, this could, e.g., have the consequence that the free energy barrier for producing gas phase hydrogen molecules is lowered.

The results for ammonia production on a partially oxidized molybdenum nanocluster indicates that the formation of ammonia on the molybdenum nanocluster is possible at a low onset potential, but with a low Faradaic efficiency due to the parasitic formation of both adsorbed H or OH on Mo_13_O_9_ and Mo_13_O_6_. Adsorption of nitrogen seems to hinder the further reduction of OH at lower oxygen coverages. This indicates that the nanocluster should be fully reduced, i.e., no oxygen present on the surface, before nitrogen is able to reach the catalyst surface. Small amounts of oxygen present at the molybdenum nanocluster surface, arising from either the electrolyte or from the preparation of the electrocatalyst, should therefore not affect the electrochemical production of ammonia.

## Conclusion

Density functional theory calculations have been employed to investigate the adsorption and reduction of oxygen on molybdenum nanoclusters. The computational hydrogen electrode was used to determine potentials for reduction of nitrogen and oxygen and the hydrogen evolution reaction.

First, a partially nitrogen covered molybdenum nanocluster was exposed to gaseous water, showing preferential adsorption of oxygen atoms compared to both hydrogen atoms and nitrogen molecules at neutral bias (*U* = 0 V), while the adsorption energies are comparable at the potential needed to produce ammonia (*U* = −0.6 V). The consequence is that the presence of water will lead to preferential oxygen adsorption at the nitrogen vacancy sites unless a negative bias is applied. The reduction of the oxygen atoms at the nitrogen rich molybdenum nanocluster was studied, and potentials more negative than −1.25 V is required to reduce the oxygen atoms into water. The main challenge is the second protonation step, where the reaction step is very dependent on the local environment. The oxygen atom will bind to the vacancy site blocking the adsorption of nitrogen molecules and thereby greatly reduce the efficiency of the nitrogen rich molybdenum nanocluster as an electro catalyst for ammonia production through the associative mechanism. A direct reduction of the nitrogen skin will, however, still be possible at *U* = −0.6 V [2].

A clean molybdenum nanocluster in contact with oxygen, hydrogen and nitrogen will preferentially form an oxygen skin at neutral bias and nitrogen adsorption on an oxygen covered molybdenum nanocluster is found to be impossible. The reduction of an oxygen overlayer was therefore studied and it was found that the reduction requires potentials of −0.29 V to −0.72 V to successfully produce water from adsorbed oxygen atoms.

For oxygen coverages of nine or less oxygen atoms, adsorption of nitrogen and hydrogen becomes possible. The electrochemical production of ammonia for adsorbed nitrogen molecules at partial oxygen coverage will only require potentials of −0.45 V to −0.7 V to make the reaction exergonic. These onset potentials are similar to values reported in earlier studies of electrochemical ammonia production on molybdenum nanocluster with or without a nitrogen skin [2]. At the potentials needed to make the ammonia production exergonic, hydrogen is found to be present on the surface, and a reduction of oxygen to OH is observed, before electrochemical production of ammonia is possible.

On the basis of the work presented here, we propose that (partially) oxidized and nitrided molybdenum nanoclusters can be re-activated for ammonia production by electrochemical reduction of the adsorbed oxygen resulting from, e.g., the catalyst synthesis procedure or the presence of water in the electrolyte, at a potential of approximate −0.7 V. This makes molybdenum nanoclusters a highly promising catalyst for electrochemical ammonia production via the associative mechanism.

## Supporting Information

File 1 Additional Figures.
