# Supplementary material for: The role of oxygen and water on molybdenum nanoclusters for electro catalytic ammonia production
Source: Beilstein J Nanotechnol. 2014 Jan 31;5:111–20. doi: 10.3762/bjnano.5.11 (PMC3943626; doi:10.3762/bjnano.5.11)
Supplement: File 1 — Additional Figures. [file Beilstein_J_Nanotechnol-05-111-s001.pdf]

Supporting Information

for

The role of oxygen and water on molybdenum nanoclusters for electro catalytic ammonia production

Jakob G. Howalt[1,2] and Tejs Vegge*[1]

Address: [1]Department of Energy Conversion and Storage, Technical University of Denmark, DK-4000 Roskilde, Denmark and [2]Center for Atomic-scale Materials Design, Department of Physics, Technical University of Denmark, DK-2800 Kgs. Lyngby, Denmark

Email: Tejs Vegge - teve@dtu.dk

*Corresponding author

Additional Figures

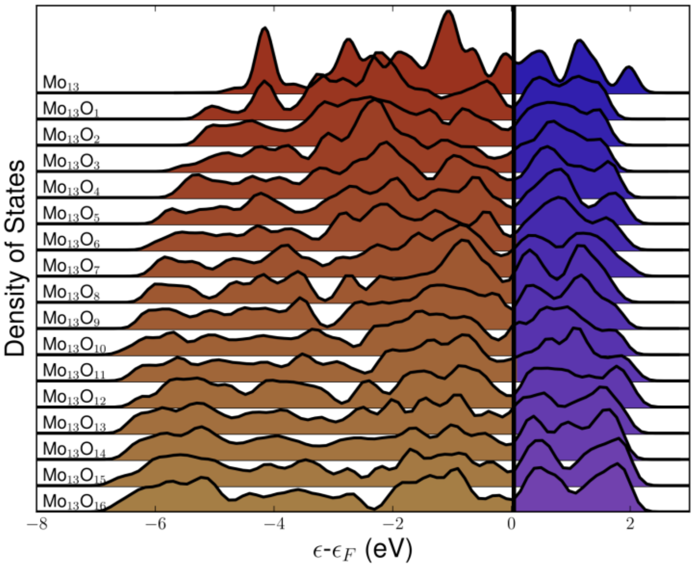

Figure S1: The d-band of molybdenum nanoclusters as the oxygen coverage evolves.

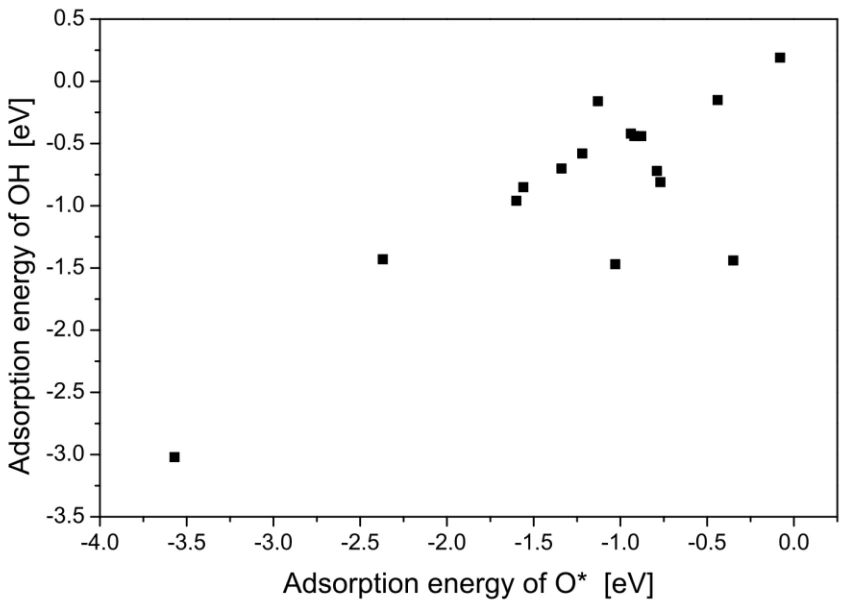

Figure S2: The adsorption energy of OH plotted as a function of adsorption energy of O for the oxygen covered molybdenum nanoclusters.

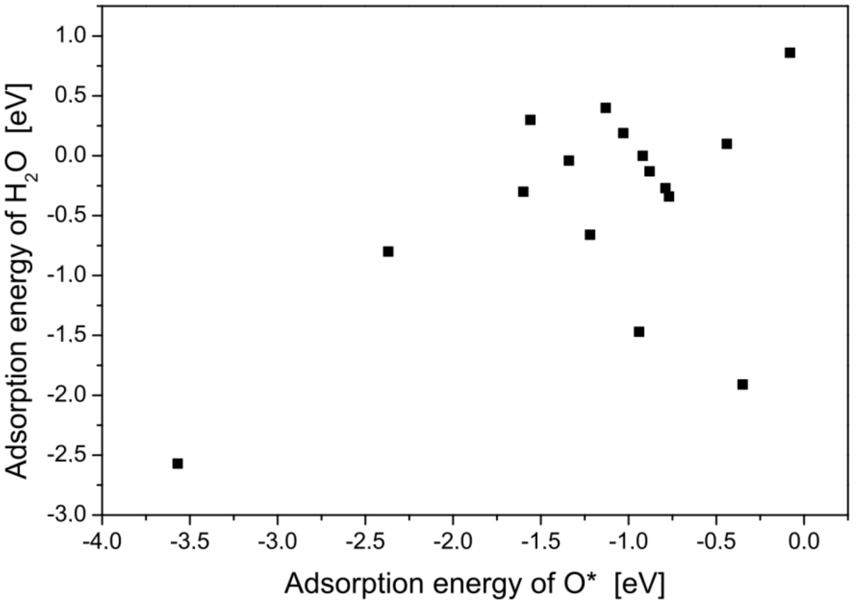

Figure S3: The adsorption energy of H_2O plotted as a function of adsorption energy of O for the oxygen covered molybdenum nanoclusters.
